# Do Dogs (*Canis lupus familiaris*) Make Counterproductive Choices Because They Are Sensitive to Human Ostensive Cues?

**DOI:** 10.1371/journal.pone.0035437

**Published:** 2012-04-25

**Authors:** Sarah Marshall-Pescini, Chiara Passalacqua, Maria Elena Miletto Petrazzini, Paola Valsecchi, Emanuela Prato-Previde

**Affiliations:** 1 Psychology, Department of Biomedical Sciences and Technologies, University of Milan, Milan, Italy; 2 Department of Evolutionary and Functional Biology, University of Parma, Parma, Italy; Hungarian Academy of Sciences, Hungary

## Abstract

Dogs appear to be sensitive to human ostensive communicative cues in a variety of situations, however there is still a measure of controversy as to the way in which these cues influence human-dog interactions. There is evidence for instance that dogs can be led into making evaluation errors in a quantity discrimination task, for example losing their preference for a larger food quantity if a human shows a preference for a smaller one, yet there is, so far, no explanation for this phenomenon. Using a modified version of this task, in the current study we investigated whether non-social, social or communicative cues (alone or in combination) cause dogs to go against their preference for the larger food quantity. Results show that dogs' evaluation errors are indeed caused by a social bias, but, somewhat contrary to previous studies, they highlight the potent effect of stimulus enhancement (handling the target) in influencing the dogs' response. A mild influence on the dog's behaviour was found only when different ostensive cues (and no handling of the target) were used in combination, suggesting their cumulative effect. The discussion addresses possible motives for discrepancies with previous studies suggesting that both the intentionality and the directionality of the action may be important in causing dogs' social biases.

## Introduction

Recent research in the infant literature has shown the power of specific ‘ostensive signals’ i.e. cues such as direct gazing, motherese and use of the child's name and body orientation in assisting social learning ([1]–[3] and [4] for a recent review). The demonstrator's ostensive cues and/or actions perceived as ‘intentional’ have also been shown to lead to inefficient choices in young children. For example children ‘over-imitate’, i.e. copy redundant actions [2]–[6] and carry out ‘obvious’ errors in their decision making process [7], when ostensive cues are being used, showing that reliance on social information may not always be the most adaptive strategy [8], [9].

It has been proposed that dogs, potentially uniquely amongst non-human species, also respond to human ostensive cues in similar contexts and support has been gathering from various studies. For example, dogs were shown to be faster at socially learning a detour task when the demonstrator talked and looked at them during the demonstration [10]; furthermore, ostensive cues are thought to have played a role in dogs' selective imitation of a conspecific's action ([11] but see [12] for an alternative explanation of the imitative effect). However, more recent studies have been less consistent, with one study showing no preferential imitation of bodily actions in a communicative context [13] and another finding a hindering effect of communicative signals in the social acquisition of an object manipulation task [14].

Like in children, also in dogs there is some indication of various kinds of evaluation errors caused by a greater reliance on socially acquired rather than individually available information. In one study [15] dogs were found to rely more on a human pointing gesture than on their own olfactory abilities when attempting to obtain hidden food, whereas another study found that dogs would select a visibly non-baited container more after a communicative than a non-communicative demonstration by the experimenter, if the experimenter then stayed in the dog's presence [16]. Finally, dogs that preferentially choose the larger of two food quantities when alone, can be induced to lose that preference when their owner (or a stranger [17]) vocally and behaviourally shows an interest for the smaller food quantity [18]. In the latter studies the owner and stranger used ostensive cues, such as gaze-alternation and a high pitched voice, to express their interest for the smaller food quantity, suggesting that these behaviours may have been responsible for the dogs' evaluation errors.

Ostensive cues have also been used in the classic A-not-B paradigm, where infants and dogs have to choose between two locations after observing the demonstrator hiding a toy behind one of them. Dogs, like infants, have been shown to be sensitive to the use of ostensive signals (voice, gaze alternation and movement), in that more perseverative errors were carried out when such signals were observed (social-communicative trials) than when they were not (non-communicative trials) [19]. However, this point is still a matter of debate since in this particular study it was difficult to disentangle the effects of enhancing a stimulus through non-social (e.g. movement/sound of the target alone) vs. social (presence of a person) vs. communicative (the person manifests specific cues) means [20], [21]. Furthermore, a combination of communicative cues (calling the dogs' name and looking at it whilst holding up the ball) were presented against a single non-social cue (holding the ball and making it squeak), hence it was not possible to disentangle whether it was the communicative nature of the stimuli or the intensity of the combined behaviours which attracted the dogs' attention more and hence affected their subsequent choice.

In a number of previous studies, it appeared difficult to tease apart the influence of communicative cues and other social mechanisms such as stimulus and local enhancement. Stimulus enhancement occurs when an animal directs its behaviour towards an object [22], [23], while local enhancement occurs when an animal directs its behaviours towards the place in which it witnessed another individual act [24], [25]. Together these mechanisms can be considered simple forms of social enhancement, which have been shown to be widespread and potentially very powerful in many animal species [25], [26].

Hence in the current study we used the experimental paradigm adopted in Prato-Previde et al. [18] to: 1. ascertain whether the evaluation error (i.e. loss of preference for the larger food quantity when the experimenter shows interest in the smaller one) observed in previous studies was due to social enhancement, 2. whether it would be elicited only with the presentation of ostensive communicative cues and 3. if so which communicative cues alone or in combination would influence dogs more strongly.

In the current paradigm a number of elements were considered to potentially play an important role in influencing the dogs' behaviour, i.e. the presence/absence of a model approaching the target; the hand-to-mouth action with or without hand-food contact; the use of voice and gaze alternation; and the combination of elements outlined above. Accordingly, dogs were allocated to one of nine groups differing in the presence/absence of specific social and communicative cues: 1. Independent choice- dogs were presented with a discrimination task between large and small quantity of food with no human intervention; this group formed the baseline against which all other groups could be compared; 2. Non-social enhancement- the target stimulus was enhanced in a non-social manner (by lifting remotely); 3. Local enhancement- the (human) model approached the target stimulus; 4. Stimulus enhancement- the model approached and picked the target stimulus up bringing it to the mouth (hand-food contact); 5. Ostensive enhancement- the model approached the target picking the food up, gaze alternating and talking to the dog; 6. Voice- the model approached the target and talked with a high-pitched tone of voice without looking at the dog; 7. Gaze alternation- the model approached the target and alternated her gaze between it and the dog; 8. Voice+Gaze- as above combining both communicative cues; 9. Hand-to-mouth- the model approached the target moved the hand from target to mouth but without making contact with the food.

Prato-Previde et al.'s experimental paradigm is particularly well suited for testing the relative weight of social vs. ostensive cues because in the crucial condition it places dogs in a visibly conflicting situation, where a valuable resource (the large food quantity) is placed in direct opposition with the social/communicative behaviours exhibited by the person choosing the less valuable resource (small food quantity).

If dogs are not affected by the cues presented to them, we expect either an improvement (due to learning/practice) or no change in the preference for the larger food quantity from the trials in which dogs choose independently (no influence condition) to trials with a demonstration (counterproductive influence condition), whereas, if the cues presented influence the dogs' choices we expect a drop in the preference for the larger food quantity, as observed in previous studies [17], [18]. Thus, following the person comes at a cost. The more powerful the cues exhibited the more willing the dogs should be to ignore their own independent source of information and go with the social information instead.

If, as has been suggested, ostensive cues are particularly salient for domestic dogs, then we would expect dogs in the communicative cues groups (Voice, Gaze, Voice+Gaze and Ostensive enhancement) to lose their preference for the larger food quantity in the crucial counterproductive condition more than dogs in our independent choice group. If however the evaluation error shown by dogs is due to a simpler social enhancement effect then we would expect dogs in the stimulus enhancement and local enhancement group to also show a loss of preference compared to the independent choice group. Finally, if the effect is not linked to social factors at all, but any kind of attention-getting cues (e.g. the food swinging and falling on the plate with an audible plop) are enough to confuse dogs and make them loose their preference for the large food quantity, then we would expect a loss of preference for the large food quantity also in the non-social enhancement group.

## Methods

### Ethics Statement

No special permission for use of animals (dogs) in such socio-cognitive studies is required in Italy. The relevant ethical committee is the Ethical Committee of the Università degli Studi di Milano.

### Subjects

149 dog-owner dyads were recruited through personal contacts, advertisements in parks and veterinary surgeons. The dog sample consisted of 60 males and 89 females whose ages ranged from 1 to 10 years (mean = 4.4 years, SD = 3.3). One hundred and two dogs were pure-breed (see Text S1) and 47 mixed-breed. All the dogs were kept for companionship, lived within the human household and had either no or only basic training experience. A number of dogs had participated in other studies by our group but not in studies using the experimental paradigm adopted here. Dogs were semi-randomly allocated to one of nine groups, counterbalancing as much as possible for age, sex and participation in other studies.

### Procedure

**Figure 1:**
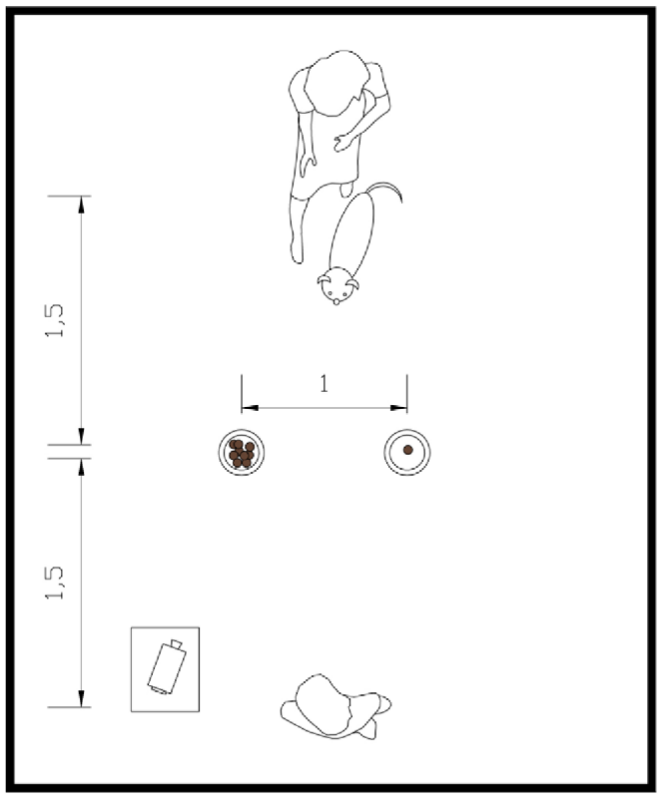
Schematic depiction of the experimental setup.

All testing took place in a relatively bare testing room at the ‘*Canis sapiens*’ laboratory of the University of Milan. The behaviour of the dog and its owner during testing was video-recorded using a wide-angle video camera positioned on a tripod located in one corner of the testing area (Figure 1).

Prior to testing the owner was asked to enter with his/her dog into the testing room and the dog was allowed to freely explore the environment whilst the experimenter described the procedure to the owner. In order to be sure that the dogs would be sufficiently motivated to perform the food choice task the owners were asked not to feed their dogs at least four hours prior to testing. In addition, the palatability of the food used was always evaluated by offering the dog a few pieces prior to testing.

The food was presented in two plastic dishes (22 cm in diameter×2 cm). Two different quantities of food were used: small, consisting of a single piece of food, and large, consisting of six pieces of food evenly spread out on the plate.

The procedure consisted of three conditions: Condition 1- **Neutral influence**: the choice between the two equally small quantities (one piece of food in each plate) with the demonstrator showing an interest for either one or the other; Condition 2- **No influence**: dogs can choose between the large and small food quantity with no person influence (this condition was identical for all groups); and Condition 3- **Counterproductive influence**: the choice between the large and small food quantity with the demonstrator showing an interest for the small one.

Dogs received a total of 18 trials, i.e. six trials in each condition. The position of the large and small food quantity was counterbalanced both in Condition 2 and Condition 3. To avoid the development of a side preference the same food quantity was never placed in the same location more than twice in a row. In Condition 1 the position of the demonstrator was counterbalanced in the same manner. In all conditions the dogs were on a leash held by their seated owners 1.5 m from the axis on which the plates were set. The plates were set 1 m apart equally distant from the dog (Figure 1).

In most conditions (apart from the non-social enhancement group- see below) the experimenter started the procedure by taking two steps towards the dog, crouching and simultaneously placing the plates on the ground in their specified positions. After exhibiting the communicative signals appropriate to the group condition (see below) the experimenter took two steps back reaching a central position between the plates and with her back turned to the dog. Once in position the experimenter told the owner to drop the leash, allowing the dog to make its choice. After the dog had chosen one of the two plates, the other was quickly removed by the experimenter.

Dogs were allocated to one of the following groups (see Movie S1 for demonstrations of procedures for each group):

#### Group 1- Independent choice (learning)

Having placed the plates on the ground the experimenter took up the neutral back-turned position between the two plates. Thus, dogs were presented with the large and small quantity with no interference from the experimenter, for a total of 12 trials. This group allows us to assess whether with no interference from presented cues, dogs will show an improvement or no change in their choice of the larger food quantity between the first six vs. latter six trials.

#### Group 2- Non-social enhancement (no person visible)

A person hidden behind a 126×78 cm screen manipulated the presentation of the plates (sliding them simultaneously in front of the dog from behind the panel) and the enhancement of the target piece of food which was lifted and then dropped with an audible ‘plop’ on the plate by using a ‘fishing rod’. Thus throughout this condition dogs never saw the person although the timing of the presentation was the same as for other groups.

#### Group 3- Local enhancement (approach only)

Having placed the plates on the ground the experimenter stood up, took a step towards one of the plates, and crouched down looking intently at the food for 5 seconds. She then stood up again and took up the neutral back-turned position between the two plates.

#### Group 4- Stimulus enhancement (approach+hand-food contact)

Having placed the plates on the ground the experimenter stood up, took a step towards one of the plates, and crouched down. Whilst looking at the plate, the experimenter picked up the food bringing it level to the mouth and holding it there for 5 seconds. She then placed the food back down, stood and took up the neutral back-turned position between the two plates.

#### Group 5- Ostensive enhancement (approach+hand-food contact+voice+gaze-alternation)

Having placed the plates on the ground the experimenter stood up, took a step towards one of the plates, and crouched down again, picked up the food bringing it level to her mouth and looking intently first at the food then at the dog then at the food again, said with a friendly tone of voice “Oh wow, this is good, this is so good!” (for the total duration of 5 seconds). She then stood and took up the neutral back-turned position between the two plates.

#### Group 6- Voice (approach+voice)

Having placed the plates on the ground the experimenter stood up, took a step towards one of the plates, and crouched down looking intently at the food for 5 seconds whilst saying in a friendly tone of voice “Oh wow, this is good, this is so good!”. She then stood and took up the neutral back-turned position between the two plates.

#### Group 7- Gaze alternation (approach+gaze-alternation)

Having placed the plates on the ground the experimenter stood up, took a step towards one of the plates, and crouched down looking intently first at the food then at the dog then at the food again, for the duration of 5 seconds. She then stood and took up the neutral back-turned position between the two plates.

#### Group 8- Voice+Gaze alternation (approach+voice+gaze-alternation)

Having placed the plates on the ground the experimenter stood up, took a step towards one of the plates, and crouched down looking intently first at the food then at the dog then at the food again whilst saying in a friendly tone of voice “Oh wow, this is good, this is so good!” (total duration being 5 seconds). She then stood and took up the neutral back-turned position between the two plates.

#### Group 9- Hand-to-mouth (approach+hand-to-mouth movement)

Having placed the plates on the ground the experimenter stood up, took a step towards one of the plates, and crouched down again looking intently at the food for 5 seconds whilst her open and visibly empty hand moved from the plate to her mouth. She then stood and took up the neutral back-turned position between the two plates.

**Figure 2:**
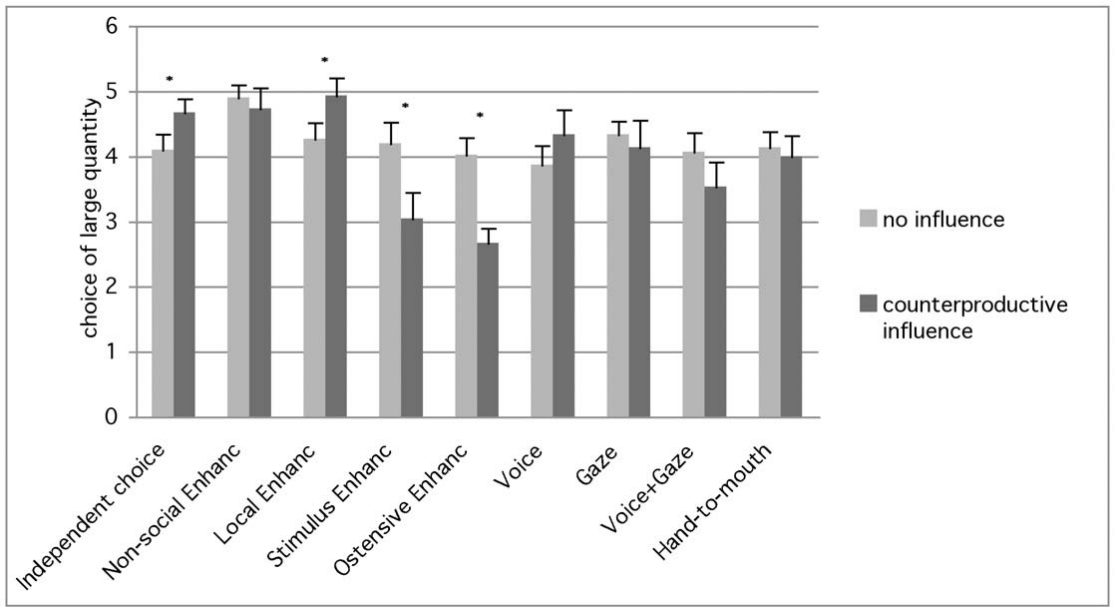
Dogs' choice of the large food quantity. Mean choice (and SEM) of the large food quantity in the no influence and counterproductive influence condition for each group (significant differences are shown for within group comparison *P<0.01). In the counterproductive influence condition dogs in the Ostensive enhancement and Stimulus enhancement group chose the larger quantity significantly less than dogs in the Independent choice group.

### Data analysis

The dogs' choices were scored from video. Only dogs that were placed in a central position between the plates and had looked at both plates before being released were included in the analysis. We recorded the number of times a dog chose the large food quantity in Condition 2 and 3 and the number of times it followed the demonstrator's indication in Condition 1 (i.e., dogs scored 0 to 6 in each condition).

Furthermore, the dog's gazing behaviour whilst being held by the owner and prior to making a choice was coded using the Solomon (beta 091110, Copyright 2006–2008 by András Péter) program. In Condition 1 and 3, whilst the experimenter was expressing her choice of plates we recorded the time spent by the dog looking at 1. the plate chosen by the experimenter, 2. the other (non-chosen) plate and 3. elsewhere. The dog's looking time was recorded from the moment the person placed the plates on the ground, to the moment the dog was released. A looking index was computed with (time spent looking at the target plate)/(time spent looking at the target plate+the other plate)*100, over the 6 trials in each condition.

For both the gazing behaviour and the dogs choice 20% of the data was coded by a second observer. Reliability for choice was 100%; reliability for the duration of gazing were all above α = 0.76 (Cronbach's alpha).

To assess whether dogs in each group showed a preference for the larger food quantity in the no influence condition (Condition 2) a One-sample Wilcoxon test was used. A Generalized Linear model (Poisson distribution) was run to compare groups in Condition 1 (Neutral influence), with number of times the dogs followed the person as the dependent variable and group as the factor. Furthermore, a Generalized Estimating Equation and post-hoc pairwise comparisons with Bonferroni correction were used to assess the between and within group differences in the dogs' preference for the larger quantity in Condition 2 (No influence) and Condition 3 (Counterproductive influence). The dependent variable was the number of times dogs chose the larger food quantity; the ‘independent choice’ group was set as the control group; factors were group and condition and both main effects and interactions were calculated.

Finally, a Generalized Estimating Equation (with Linear distribution) and post-hoc comparisons with Bonferroni correction were also used to assess the between and within group differences in the dog's looking time to the target/person's plate in the neutral influence (Condition 1) and counterproductive influence (Condition 3). The dependent variable was the looking time index, the factors were group and condition and both main effects and interactions were calculated.

Statistical tests were two-tailed, the α value was set at 0.05 and the statistical package SPSS version 19 was used.

## Results

Twelve of the 149 dogs tested were removed from analysis either because over the 18 trials they revealed a 100% side bias or because they did not complete all 18 trials. All dogs looked at both plates prior to choice. This resulted in the following group composition: Group 1- Independent choice: 14 dogs (7 F, 7 M); Group 2- Non-social enhancement: 15 dogs (10 F, 5 M); Group 3- Local enhancement: 15 dogs (9 F, 6 M); Group 4- Stimulus enhancement 14 dogs (9 F, 5 M); Group 5- Ostensive enhancement: 15 dogs (9 F, 6 M); Group 6- Voice: 17 dogs (11 F, 6 M); Group 7- Gaze alternation: 17 dogs (9 F, 8 M); Group 8- Voice+Gaze alternation: 15 dogs (8 F, 7 M); Group 9- Empty hand-pick: 15 dogs (11 F, 4 M). Looking time was analysed only for dogs who had witnessed a demonstration (thus excluding the independent choice group): however, due to video malfunction only 116 of the 123 dogs could be analysed for looking time, thus results are based on this data.

Overall, in Condition 2 (no influence) 100 (73%) dogs chose the larger food quantity more often (i.e. on either four or more trials out of the total six); 31 (23%) dogs chose at random (i.e. chose the larger food quantity on three out of six trials), 6 (4%) dogs chose the larger food quantity twice or less. Thus overall, dogs significantly preferred choosing the larger food quantity (One-sample Wilcoxon: Group 1: z = 2.63, p = 0.02; Group 2: z = 3.43, p<0.001; Group 3: z = 2.97, p = 0.001; Group 4: z = 2.62, p = 0.02; Group 5: z = 2.38, p<0.01; Group 6: z = 3.64, p<0.001; Group 7: z = 3.63, p<0.001; Group 8: z = 3.45, p<0.001; Group 9: z = 3.43, p<0.001).

No differences between groups emerged in the dogs' choice of the target plate in Condition 1 (Neutral influence) (Wald = 7.75; df = 7, p = 0.35). However, the generalized estimating equation comparing all groups in Condition 2 vs. Condition 3, with the choice of the large food quantity as dependent variable, revealed main effects for both group (Wald = 37.8, df = 8, p<0.001) and condition (Wald = 3.8, df = 1, p = 0.051) as well as a significant interaction between the two (Wald = 84.8, df = 8, p<0.001), with the Ostensive, Stimulus enhancement and Voice+Gaze group, showing a significantly different performance from the baseline independent choice group in Condition 3 (Ostensive p<0.001; Stimulus p<0.001; Voice+Gaze p = 0.01) but not in Condition 2 (Ostensive p = 0.99; Stimulus p = 0.47; Voice+Gaze p = 0.65) (Figure 2).

Post-hoc analyses were also carried out to assess the change in preference for the large food quantity between Condition 2 (No influence) and Condition 3 (Counterproductive influence) in each group. A significant difference emerged in the Independent choice (p = 0.007), Local enhancement (p = 0.003), Stimulus enhancement (p = 0.009) and Ostensive cues (p = 0.001) group with dogs in the first two groups showing an improvement in performance from Condition 2 to Condition 3, and dogs in the latter two groups showing a drop in their preference for the larger food quantity (Figure 2).

**Figure 3:**
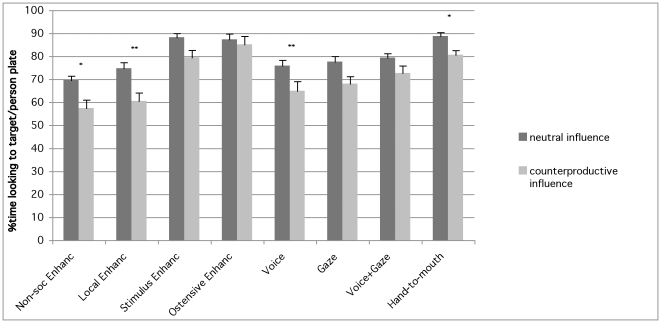
Looking index. Mean (and SEM) durations (in percentage) of looking to the target/experimenter plate in the neutral choice and counterproductive influence condition for each group (significant differences are shown for within group comparison *P<0.05, **P<0.01; see Tables 1 and 2 for between group results).

As regards looking time, a main effect of both group (Wald = 113.96, df = 7, p<0.001) and condition (Wald = 78.22, df = 7, p<0.001) emerged as well as an interaction between the two factors (Wald = 17.66, df = 7, p = 0.01). Post-hoc analyses showed that dogs looked at the person/target plate more in the Neutral than in the Counterproductive condition in the Non-social enhancement (p = 0.04), Local enhancement (p = 0.003), Voice (p = 0.001) and Hand-to-mouth group (p = 0.02), whereas no such difference emerged in the Stimulus enhancement (p = 0.14), Ostensive enhancement (p = 1), Gaze (p = 1) and Voice+Gaze (p = 1) group. Furthermore, in Condition 1 dogs in the Ostensive, Stimulus enhancement and Hand-to mouth group looked at the person/target plate more than dogs in most other groups (Table 1 and Figure 3). In the Counterproductive condition however, dogs in the Ostensive group continued to look at the person as much as dogs in the Stimulus enhancement and Hand-to-mouth group, but whereas dogs in the Ostensive group differed from all other groups, dogs in the Stimulus and Hand-to-mouth group continued to look more at the person than dogs only in the Non-social and Local enhancement groups (Table 2 and Figure 3).

**Table 1:** Looking at the target/person plate in the Neutral condition (Condition 1) for dogs in each group.

	non-soc enh	local enh	stim enh	ost enh	voice	gaze	voice+gaze	hand-to-mouth
mean in %	69.59	74.74	88.31	87.31	75.95	77.66	79.37	88.76
**non-soc enh**		1	<0.001	<0.001	1	0.964	1	<0.001
**local enh**	1		0.001	0.007	1	1	1	0.001
**stimulus enh**	<0.001	0.001		1	0.003	0.03	0.2	1
**ostensive enh**	<0.001	0.007	1		0.03	0.2	0.9	1
**voice**	1	1	0.003	0.03		1	1	0.004
**gaze**	1	1	0.03	1	1		1	0.03
**voice+gaze**	0.11	1	0.2	0.92	1	1		0.2
**hand-to-mouth**	<0.001	0.001	1	1	0.004	0.03	0.2	

Results (p-values) of the between group post-hoc comparisons (with Bonferroni corrections).

**Table 2:** Looking at the target/person plate in the Counterproductive condition (Condition 3) for dogs in each group.

	non-soc enh	local enh	stim enh	ost enh	voice	gaze	voice+gaze	hand-to-mouth
mean in %	57.46	60.55	79.53	85.16	65.01	68.07	72.73	80.59
**non-soc enh**		1	<0.001	<0.001	1	1	0.1	<0.001
**local enh**	1		0.006	<0.001	1	1	1	0.002
**stimulus enh**	<0.001	0.006		1	0.27	1	1	1
**ostensive enh**	<0.001	<0.001	1		<0.001	0.01	0.04	1
**voice**	1	1	0.27	<0.001		1	1	0.1
**gaze**	1	1	1	0.01	1		1	1
**voice+gaze**	0.1	1	1	0.04	1	1		1
**hand-to-mouth**	<0.001	0.002	1	1	0.1	1	1	

Results (p-values) of the between group post-hoc comparisons (with Bonferroni corrections).

## Discussion

There is currently some debate as to the extent of dog's sensitivity to ostensive cues, the mechanisms behind this phenomenon and its function in the dog-human relationship. At present it is not clear whether ostensive cues influence the dogs' learning process above and beyond simply attracting the dog's attention more to the demonstrated actions, in fact in a recent study, dogs were shown to learn a detour task as efficiently from a partial demonstration from an inanimate object than from a human demonstrating the action whilst calling the dog [27].

In the current study, by placing dogs in a conflict situation we were able to assess the relative weight of each communicative cue both in attracting the dog's attention (looking time) during the demonstration, and in influencing the dog's choice behaviour. The results from the looking time data show that even in the condition where a conflict between larger food quantity and demonstration exists (Counterproductive influence) dogs in all groups looked at the demonstrator for more than 57% of the trial time (with the remaining time spent looking at the other plate). However, differences between demonstrations emerged clearly: the most powerful attention-getting behaviours were not the communicative cues (neither alone nor combined), but rather the Hand-to-mouth action with our without food-contact. Yet, although dogs in the Hand-to-mouth group looked at the demonstration to the same extent as dogs in the Stimulus and Ostensive enhancement group, their choices were not affected in the same way since dogs in the hand-to-mouth group *did not* go against their preference for the larger food quantity. Interestingly, this shows that although human cues can strongly influence both aspects of the dogs' behaviour they may not necessarily do so in parallel.

In terms of the dogs' choices in the current study we investigated whether dogs would choose against their preference in a food quantity discrimination task according to the type of non-social, social and communicative cues presented.

With no interference from the demonstrator (i.e. No influence condition) dogs in all groups showed an overall preference for the larger food quantity and dogs that were allowed to continue choosing with no interference for 12 trials (Independent choice group) showed an improvement across the first and last six trials. Thus dogs, overall, prefer the larger food quantity and with practice become better at discriminating between the two.

Given that dogs in the absence of interference prefer a larger food quantity the central question is under which conditions they would choose against their preference. The same pattern of improvement in quantity discrimination found in the Independent choice group was replicated also in the Local enhancement group, thus dogs who witnessed a person approaching and squatting down next to the plate but showing no communicative cues and no handling of the food, were able to improve their choice of the larger food quantity across trials. Contrary to this pattern, dogs in the Stimulus enhancement and Ostensive enhancement groups (presented with the demonstrator either handling the food or both handling the food and looking and talking to them) started choosing *against* their preference more often giving up the larger food quantity to follow the model's choice of the smaller one. Dogs in the other groups (Non-social enhancement, Voice, Gaze, Hand-to-mouth, Voice+Gaze) showed neither a significant improvement nor a significant drop in performance.

Predictably (given results from the within group comparisons), when compared to the Independent choice group dogs in the Stimulus and Ostensive group showed a significantly lower preference for the larger food quantity in the counterproductive influence condition, however the same pattern of results emerged also for the Voice+Gaze group, suggesting that dogs in this group are also, to a certain extent, affected by the demonstration.

Given dogs did not go against their preference if they saw food being lifted and falling with no human intervention (the Non-social enhancement group) we can conclude that the evaluation error observed in the current study was caused by a social bias, which could be induced either by a highly salient social cue such as handling the food (Stimulus enhancement group) or, to a lesser extent, by using a combination of communicative cues such as talking in a friendly voice and gaze alternation (Voice+Gaze group).

Current results highlight the potent effect of stimulus enhancement (in this case handling the food) as a social influencing/learning mechanism and are somewhat at odds with other published work where stimulus enhancement unaccompanied by communicative cues did not influence the dogs' choices [16], [19], [21]. It is perhaps interesting to note however, that in both the Topal et al. studies [19], [21] and the Kupan et al. study [16] toys were used as the target object, whereas in our own study food was the source of interest. The discrepancy in results may thus be linked to the use of these different stimuli. It may be that dogs considered picking up the food (with no other signal) as a communicative cue directed at them (showing them ‘their’ food), but did not view manipulation of an object (with no other cue) as an invitation to interact with it. Dogs may be used to seeing us manipulate objects that are of no concern to them, but manipulating food from the ground may be more easily considered an explicit invitation even with no further communicative cue present. This may be supported by the fact that when the demonstrator did not pick the food up (thus only local enhancement was used) dogs were able to totally ignore the experimenter's behaviour.

Another possibility is that the perception of the other's action as intentional may be in some cases sufficient to produce a social bias even when no communicative cue indicates to whom the action is directed to. Infants of a very young age have been shown to perceive grasping an object as an intentional action [28], [29] and evidence from mirror neuron studies in macaques also suggest that grasping is perceived as goal-directed [30]. Furthermore, a recent study showed that children's ‘over-imitation’ (copying obviously useless actions) is linked to the perceived intentionality of the demonstrator's actions [6]. In our study the same hand movement (hand-to-mouth) with or without the grasping of the target, produced similar levels of looking/attention but very different effects on the dogs' behaviour. Only in the latter case did dogs go against their preference. It is thus possible that whereas both demonstrations attracted the dogs' attention only the grasping motion was perceived as intentional and that this may play a role in causing the observed social bias. This would be in line with results showing that the intentionality of a pointing gesture can be discerned and may be sufficient to affect dogs' choices in a classic two-choice task [31].

Thus, what seems to emerge from previous studies and our own is that the dog's social bias may be influenced by a combination of the perception of the intentionality behind the action and the communicative framework within which it is placed. In line with other studies [31] we found that the latter appears to be perceived the strongest when communicative cues are used in combination (voice+gaze) rather than singularly, most probably since this reflects the naturalistic interactions between dogs and humans. A further element, which may be important in the emergence of a social bias is the dog's motivation to interact with the stimuli (i.e. the potential differences of food vs. toys). Future studies will need to consider these aspects separately to tease apart the relative importance of each.

Finally, current results have interesting implication for the hypothesis that social learning may in some cases be maladaptive. A recent review [9] highlighted the need for studies which would address the potential occurrence of “informational cascades”, where suboptimal methods may spread rapidly across a population because social information comes to outweigh personal experience, however very few studies have explored under what conditions an animal will choose to rely on private vs. social information [8], [9], [32]–[36]. The current study adds to a small but growing literature showing that social learning is not necessarily always the best strategy [34]–[39] and provides an experimental paradigm which may potentially be used to explore when an animal will rely on private vs. social information.

## Supporting Information

Movie S1 **Videos of the demonstrations witnessed by dogs in each group.**
(WMV)

Text S1 **Breeds of participating dogs.**
(DOC)
