# Supplementary material for: Do Dogs (Canis lupus familiaris) Make Counterproductive Choices Because They Are Sensitive to Human Ostensive Cues?
Source: PLoS One. 2012 Apr 25;7(4):e35437. doi: 10.1371/journal.pone.0035437 (PMC3338840; doi:10.1371/journal.pone.0035437)
Supplement: Text S1 — Breeds of participating dogs. (DOC) [file pone.0035437.s002.doc]

**Breeds of participating dogs**

3 American Staffordshire, 2 Argentinean dogo, 1 Azawak, 2 Basenji, 3 Beagle, 1 Belgian shepherd, 1 Bichon, 5 Border collie, 3 Boxer, 2 Brittany spaniel, Chihuahua, Cocker spaniel, Czechoslovakian wolfdog, 2 Dachshund, 1 Doberman, 2 English setter, 3 Flatcoated retriever, 3 German shepherd, 1 Giant poodle, 7 Golden retriever, 1 Great dane, 1 Hovawart, 1 Hungarian vizla, 1 Italian hound, 1 Italian lagotto romagnolo, 1 Italian spitz, 1 Istrian shorthaired hound, 4 Jack Russell, 1 Kurzhaar, 5 Labrador retriever, 3 Maltese, 2 Poodle, 2 Pug, 3 Rottweiler, 2 Scottish collies, 2 Shiba Inu, 2 Siberian husky, 2 Whippet.
